# Supplementary material for: Research on government subsidy strategy of biomass power supply chain considering channel encroachment
Source: PLoS One. 2025 May 27;20(5):e0322782. doi: 10.1371/journal.pone.0322782 (PMC12112409; doi:10.1371/journal.pone.0322782)
Supplement: S1 File — (DOCX) [file pone.0322782.s001.docx]

**Appendix 1 Derivation process**

**Proofs of Scenarios NN, NF, NM and NP**

Using the equality $\pi_{M}=\left( {p_{1}-w}_{1}-c_{2} \right)d_{1}$, we have $\frac{\partial^{2}\pi_{M}}{\partial p_{1}^{2}}=-2<0$, which shows that $\pi_{M}$ is concave in $p_{1}$. From $\frac{\partial\pi_{M}}{\partial p_{1}}=0$, we get the reaction function $p_{1}=\frac{a+c_{2}+w_{1}}{2}$. Substituting $p_{1}=\frac{a+c_{2}+w_{1}}{2}$ into the equality $\pi_{F}=\left( w_{1}-c_{1} \right)d_{1}$, we have $\frac{\partial^{2}\pi_{F}}{\partial w_{1}^{2}}=-4<0$, which shows that $\pi_{F}$ is concave in $w_{1}$. From $\frac{\partial\pi_{F}}{\partial w_{1}}=0$, we have $w_{1}=\frac{1}{2}\left( 3a+c_{1}-c_{2} \right)$. Substituting $w_{1}=\frac{1}{2}\left( 3a+c_{1}-c_{2} \right)$ into the aforementioned $p_{1}=\frac{a+c_{2}+w_{1}}{2}$, we obtain the suitable outcomes for Scenario NN.

Using a similar approach, we can obtain the equilibrium result for Scenarios NF, NM and NP, and the proof is omitted here.

**Proofs of Scenarios EN, EM, EF and EP**

Using the equality $\pi_{M}=\left( {p_{1}-w}_{1}-c_{2} \right)d_{1}$, we have $\frac{\partial^{2}\pi_{M}}{\partial p_{1}^{2}}=-2<0$, which shows that $\pi_{M}$ is concave in $p_{1}$. From $\frac{\partial\pi_{M}}{\partial p_{1}}=0$, we get the reaction function $p_{1}=\frac{1}{2}\left( a+c_{2}+bp_{2}+w_{1} \right)$. Substituting $p_{1}=\frac{1}{2}\left( a+c_{2}+bp_{2}+w_{1} \right)$ into the equality $\pi_{F}=d_{1}\left( w_{1}-c_{1} \right)+d_{2}\left( p_{2}-c_{0} \right)$, we have $\frac{\partial^{2}\pi_{F}}{\partial w_{1}^{2}}=-1<0$, $\frac{\partial^{2}\pi_{F}}{\partial p_{2}^{2}}=b^{2}-2$, $\frac{\partial^{2}\pi_{F}}{\partial w_{1}\partial p_{2}}=b$ and $\frac{\partial^{2}\pi_{F}}{\partial p_{2}\partial w_{1}}=b$. Thus, the Hessian matrix is positive, which shows that $\pi_{F}$ is concave in $w_{1}$ and $p_{2}$. From $\frac{\partial\pi_{F}}{\partial w_{1}}=0$ and $\frac{\partial\pi_{F}}{\partial p_{2}}=0$, we have $w_{1}=\frac{-a-c_{1}+bc_{1}+c_{2}-bc_{2}}{2\left( -1+b \right)}$ and $p_{2}=\frac{-a-c_{0}+bc_{0}}{2\left( -1+b \right)}$. Substituting $w_{1}=\frac{-a-c_{1}+bc_{1}+c_{2}-bc_{2}}{2\left( -1+b \right)}$ and $p_{2}=\frac{-a-c_{0}+bc_{0}}{2\left( -1+b \right)}$ into the aforementioned $p_{1}=\frac{1}{2}\left( a+c_{2}+bp_{2}+w_{1} \right)$, we obtain the suitable outcomes for Scenario EN.

Using a similar approach, we can obtain the equilibrium result for Scenarios EM, EF and EP, and the proof is omitted here.

**Proofs of Propositions**

**Proposition 1.** By comparing with the equilibrium results, we have: $w_{1}^{\mathrm{NF}}-w_{1}^{\mathrm{NM}}=-m$<0; $w_{1}^{\mathrm{NM}}-w_{1}^{\mathrm{NN}}=-\frac{m}{2}<0$; $w_{1}^{\mathrm{NN}}-w_{1}^{\mathrm{NP}}=0$. Therefore, $w_{1}^{\mathrm{NF}}<w_{1}^{\mathrm{NM}}<w_{1}^{\mathrm{NN}}=w_{1}^{\mathrm{NP}}$. In a similar way, we can get the rest of Proposition 1.

**Propositions 2-5.** Using a similar logic as in Proposition 1, we obtain Propositions 2-5, and the proof is omitted here.
