# Supplementary material for: Research on government subsidy strategy of biomass power supply chain considering channel encroachment
Source: PLoS One. 2025 May 27;20(5):e0322782. doi: 10.1371/journal.pone.0322782 (PMC12112409; doi:10.1371/journal.pone.0322782)
Supplement: Attachment — Submitted filename: Response to Reviewers.docx [file pone.0322782.s003.docx]

**Authors response to** **Academic Editor’s and Reviewers’ Comments on** **PONE-D-24-49884.**

On behalf of my co-authors, I would like to express our sincere gratitude for the opportunity to revise our manuscript. We also extend our thanks to the academic editor and reviewers for their insightful comments and constructive suggestions, which have been carefully incorporated into this revised version. Detailed responses to each comment from the academic editor and reviewers are provided below.

**Response to Academic Editor’s Comments**

**Answer:** Thanks for your valuable comments and suggestions. We will answer your comments one by one.

**Comment (1)**: Please ensure that your manuscript meets PLOS ONE’s style requirements, including those for file naming. The PLOS ONE style templates can be found at

https://journals.plos.org/plosone/s/file?id=wjVg/PLOSOne_formatting_sample_main_body.pdf and

<https://journals.plos.org/plosone/s/file?id=ba62/PLOSOne_formatting_sample_title_authors_affiliations.pdf>

**Answer:** We feel great thanks for your professional review work on our article. As you are concerned, there are several problems that need to be addressed. According to your nice suggestions, we have made partial corrections to our previous draft, the detailed corrections are listed below.

(i) We have changed the font size and typeface of Level 1 headings, Level 2 headings, and Level 3 headings as required.

(ii) Figure captions in the text have been formatted in bold type as requested.

(iii) The graphic files have been uploaded to the Prepress Check Analysis and Conversion Engine (PACE) to generate .tiff files, and then I inserted the generated .tiff files into my article.

**Comment (2)**: Thank you for stating the following financial disclosure:

“Project Title: “Route Planning for Fresh Agricultural Product Delivery Vehicles under Traffic Control Policies of Restricted Access,” Henan Province Scientific and Technological Research Project, 2024, No. 242102240028.”

Please state what role the funders took in the study. If the funders had no role, please state: “The funders had no role in study design, data collection and analysis, decision to publish, or preparation of the manuscript.”

If this statement is not correct you must amend it as needed.

Please include this amended Role of Funder statement in your cover letter; we will change the online submission form on your behalf.

**Answer:** Thank you for the constructive suggestions.

This study was supported by funding from the project: “Route Planning for Fresh Agricultural Product Delivery Vehicles under Traffic Control Policies of Restricted Access,” Henan Province Scientific and Technological Research Project, 2024, No. 242102240028. Professor Wang Heng is the funder of this project and a co-author of this paper. In this study, he provided methodological guidance and conducted research investigations. This has been detailed in the accompanying cover letter.

**Comment (3)**: We note that your Data Availability Statement is currently as follows: [All relevant data are within the manuscript and its Supporting Information files.]

Please confirm at this time whether or not your submission contains all the raw data required to replicate the results of your study. Authors must share the “minimal data set” for their submission. PLOS defines the minimal data set to consist of the data required to replicate all study findings reported in the article, as well as related metadata and methods (<https://journals.plos.org/plosone/s/data-availability#loc-minimal-data-set-definition>).

**Answer:** Thank you for your valuable advice. After our review, the submitted manuscript contains all the original data needed to reproduce the findings of the study. Details are as follows:

(i) The data cited in the introduction of this paper are all from existing research articles and websites and are reflected in the references.

(ii) No specific data was used in the calculation of balance results in this paper. Please refer to the appendix for the proof process. Therefore, there is no data such as mean, standard deviation, and other measurements.

(iii) In this paper, similar to the drawing part of Wang et al. [1]and Liu et al.[2], specific parameters (specified parameters have been indicated in the paper) are set by numerical simulation and input into the equilibrium result, which can be obtained by running the code in the mathematical software Mathematica.

**Comment (4)**: Please ensure that you refer to Figure 1 and 2 in your text as, if accepted, production will need this reference to link the reader to the figure.

**Answer:** Thank you for your significant reminder. Owing to our oversight into the initial draft, the references to Figure 1 and Figure 2 were not properly cited in the text. We have promptly addressed this issue and made the necessary corrections as outlined below.

These changes appear on Page 13. For your convenience, we repeat these modifications here:

*(1). “Scenario NN Fig 1(a)”: There is No channel competition, and the relevant sector does Not subsidize the supply chain members.*

*(2). “Scenario NF Fig 1(b)”: There is No channel competition, and the relevant sector subsidizes the farmers within the supply chain.*

*(3). “Scenario NM Fig 1(c)”: There is No channel competition, and the relevant sector subsidizes the middlemen in the supply chain.*

*(4). “Scenario NP Fig 1(d)”: There is No channel competition, and the relevant sector subsidizes the power plants in the supply chain.*

*(5). “Scenario EN Fig 1(e)”: Farmers opt to establish new supply channels, and the relevant sector does Not subsidize the supply chain members.*

*(6). “Scenario EF Fig 1(f)”: Farmers opt to establish new supply channels, and the relevant sector subsidizes the farmers in the supply chain.*

*(7). “Scenario EM Fig 1(g)”: Farmers opt to establish new supply channels, and the relevant sector subsidizes the middlemen in the supply chain.*

*(8). “Scenario EP Fig 1(h)”: Farmers opt to establish new supply channels, and the relevant sector subsidizes the power plants in the supply chain.*

**Fig 1. Eight scenarios of government subsidies for members of the biomass power generation supply chain under varying degrees of channel encroachment**

These changes appear on Page 15. For your convenience, we repeat these modifications here:

*As illustrated in Fig. 2, the sequence of decision-making follows these steps: (1) Farmers determine whether to engage in encroachment; (2) The government selects a subsidy policy. To assess the impact of decision order on equilibrium outcomes, an extension is considered where the government's subsidy decision precedes the farmer’s encroachment strategy, confirming the robustness of the main results; (3) The middleman sets the unit price for selling biomass to the power plant* $p_{1}$*; (4) Farmers determine* *the wholesale price of biomass sold to middlemen*$w_{1}$ *and the unit price of biomass sold directly to power plants* $p_{2}$ *The sequence of decisions between farmers and middlemen is detailed in the corresponding table. This decision-making framework highlights the intricate interactions and strategic competition among farmers, middlemen, and power plants. Essentially, the biomass power supply chain operates as a supply system where electricity production serves as the primary driving force* *[3]. Additionally, this section clarifies the price decision-making process corresponding to stages 3 and 4 [4].*

**Fig 2. Sequence of events**

**Response to Reviewer: 1’s Comments**

**Comment:** This study presents the strategic interaction between government subsidy strategies and farmers’ channel encroachment strategies in a biomass power supply chain. This is an interesting topic. However, some issues are provided below.

**Answer:** Thanks for your valuable comments and suggestions. We will answer your comments one by one.

**Comment (1):** Part I, Introduction and Related work seems to be logically confusing. The author should clearly indicate the main idea of each paragraph.

**Answer:** Based on your valuable comments, we have revised the first section as follows.

Firstly, we restructure the literature review into a distinct second section, separating it from the introduction. The revised organization of the first part is as follows: The initial paragraph provides an overview of the research background, emphasizing that biomass power generation is a critical component of sustainable energy development and highlighting its potential for further advancement. The subsequent paragraph elucidates that governments worldwide commonly employ policies to regulate the decisions of biomass supply chain participants, thereby fostering the sustainable development of the biomass power generation supply chain, which sets the stage for the research focus of this paper. The third paragraph underscores the pivotal role of government subsidies in guiding channel invasion within the biomass supply chain, while noting that existing literature lacks comprehensive analysis of the interplay between these two elements. The fourth paragraph delineates the primary issues addressed in this study. Finally, the fifth paragraph encapsulates the research contributions of this paper. These changes appear on Pages 2-6. For your convenience, we repeat these modifications here:

*“As conventional energy sources continue to be depleted and greenhouse gas emissions escalate, the challenges of global environmental pollution and climate change have become increasingly urgent and significant [5]. In response, governments have implemented a series of policies and regulations aimed at achieving energy conservation and emission reduction. ... The transition towards renewable energy has become an inevitable trend. Among various renewable energy sources, biomass energy, recognized as the fourth largest global energy resource, has gained prominence as a crucial approach for governments to achieve sustainable energy development due to its “carbon-neutral” characteristic [6]. Biomass energy utilization primarily includes biomass power generation as a key application [7]…The development of the biomass power generation supply chain continues to require government intervention [8] …*

*Faced with these challenges, countries typically adopt strategies to guide and regulate the decision-making behaviors of biomass supply chain participants through the formulation of relevant policies, thereby promoting the sustainable development of the biomass power supply chain [9] …However, in general, no clear and unified regulation exists concerning the recipients and distribution mechanisms of subsidies within the biomass power generation supply chain.*

*Conversely, in the biomass raw material supply sector, farmers are increasingly choosing to bypass middlemen [10] and supply raw materials directly to power plants [11] …These examples highlight the significant role of government subsidies in facilitating the expansion of farmers' supply channels. However, the interaction between government subsidies and channel expansion remains an area that has received limited scholarly attention.*

*Based on the above, we study the government’s subsidy strategy for supply chain members in the biomass straw power supply chain, it encompasses the following inquiry: …*

*This study contributes to literature in several ways … “*

**Comment (2):** Many Tables go beyond the margins.

**Answer:** We feel great thanks for your professional review of our article. As you are concerned, there are several tables that need to be adjusted.

The adjusted table is as follows：

**Table 1 .** **Comparison with the literature. （**The change appears on Page 11.**）**

|  | | **Incentive policy** | | | | | | | **Channel** | | | | | **Policy target** | | | | | | | | | | |
| --- | --- | --- | --- | --- | --- | --- | --- | --- | --- | --- | --- | --- | --- | --- | --- | --- | --- | --- | --- | --- | --- | --- | --- | --- |
|  | | **Taxes** | | | **Quota**  **system** | | **Subsidization** | | | **Encroachment** | | | **Non-encroachment** | | | | **Farmer** | | | **Middleman** | | | **Manufacturer** | |
| **[12]** |  | |  | | | **✓** | | | |  | **✓** | | | | **✓** | | |  | | |  | | |  |
| **[13]** |  | |  | | | **✓** | | | | **✓** |  | | | |  | | |  | | | **✓** | | |  |
| **[14]** |  | | **✓** | | | **✓** | | | |  | **✓** | | | | **✓** | | |  | | | **✓** | | |  |
| **[15]** | **✓** | |  | | |  | | | |  | **✓** | | | |  | | |  | | | **✓** | | |  |
| **[16]** |  | |  | | | **✓** | | | |  | **✓** | | | |  | | |  | | | **✓** | | |  |
| **[17].** |  | | **✓** | | | **✓** | | | | **✓** |  | | | |  | | | **✓** | | | **✓** | | |  |
| **[18]** |  | | **✓** | | | **✓** | | | |  | **✓** | | | | **✓** | | |  | | | **✓** | | |  |
| **[19]** |  | |  | | | **✓** | | | |  | **✓** | | | |  | | | **✓** | | | **✓** | | |  |
| **[20]** |  | | **✓** | | | **✓** | | | |  | **✓** | | | | **✓** | | | **✓** | | | **✓** | | |  |
| **[21]** |  | |  | | | **✓** | | | |  | **✓** | | | | **✓** | | | **✓** | | |  | | |  |
| **[22]** |  | |  | | | **✓** | | | |  | **✓** | | | | **✓** | | | **✓** | | | **✓** | | |  |
| **[23]** |  | |  | | | **✓** | | | |  | **✓** | | | | **✓** | | | **✓** | | | **✓** | | |  |
| **[24]** |  | |  | | |  | | | | **✓** |  | | | |  | | |  | | |  | | |  |
| **[25]** |  | |  | | | **✓** | | | | **✓** |  | | | | **✓** | | |  | | |  | | |  |
| **[26]** |  | |  | | | **✓** | | | |  | **✓** | | | | **✓** | | | **✓** | | | **✓** | | |  |
| **[27]** |  | |  | | |  | | | | **✓** |  | | | |  | | |  | | |  | | |  |
| **This paper** | |  | |  | | | | **✓** | | **✓** | | **✓** | | | | **✓** | | | **✓** | | | **✓** | |  |

**Table 3** **Equilibrium pricing in the no-encroachment channel scenarios. （**The change appears on Page 19.**）**

| **Scenario** | $\boldsymbol{w}_{\mathbf{1}}$ | $\boldsymbol{p}_{\mathbf{1}}$ |
| --- | --- | --- |
| $\mathbf{NN}$ | $\frac{1}{2}(a+c_{1}-c_{2})$ | $\frac{1}{4}(3a+c_{1}+c_{2})$ |
| $\mathbf{NF}$ | $\frac{1}{2}(a+c_{1}-c_{2}-m)$ | $\frac{1}{4}(3a+c_{1}+c_{2}-m)$ |
| $\mathbf{NM}$ | $\frac{1}{2}(a+c_{1}-c_{2}+m)$ | $\frac{1}{4}(3a+c_{1}+c_{2}-m)$ |
| $\mathbf{NP}$ | $\frac{1}{2}(a+c_{1}-c_{2})$ | $\frac{1}{4}(3a+c_{1}+c_{2})$ |

**Table 4 Equilibrium pricing in the encroachment channel scenarios. （**The change appears on Page 22.**）**

| **Scenario** | $\boldsymbol{w}_{\mathbf{1}}$ | $\boldsymbol{p}_{\mathbf{1}}$ | $\boldsymbol{p}_{\mathbf{2}}$ |
| --- | --- | --- | --- |
| $\mathbf{EN}$ | $\frac{-a-c_{1}+bc_{1}+c_{2}-bc_{2}}{2(-1+b)}$ | $\frac{\begin{aligned} -3a+ab-bc_{0}+b^{2}c_{0}-c_{1} \\ +bc_{1}-c_{2}+bc_{2} \end{aligned}}{4(-1+b)}$ | $\frac{-a-c_{0}+bc_{0}}{2(-1+b)}$ |
| $\mathbf{EF}$ | $\frac{\begin{aligned} -a-c_{1}+bc_{1}+c_{2}-bc_{2} \\ +m-bm \end{aligned}}{2(-1+b)}$ | $\frac{\begin{aligned} -3a+ab-bc_{0}+b^{2}c_{0}-c_{1} \\ +bc_{1}-c_{2}+bc_{2}+m-b^{2}m \end{aligned}}{4(-1+b)}$ | $\frac{-a-c_{0}+bc_{0}+m-bm}{2(-1+b)}$ |
| $\mathbf{EM}$ | $\frac{\begin{aligned} -a-c_{1}+bc_{1}+c_{2}-bc_{2} \\ -m+bm \end{aligned}}{2(-1+b)}$ | $\frac{\begin{aligned} -3a+ab-bc_{0}+b^{2}c_{0}-c_{1} \\ +bc_{1}-c_{2}+bc_{2}+m-bm \end{aligned}}{4(-1+b)}$ | $\frac{-a-c_{0}+bc_{0}}{2(-1+b)}$ |
| $\mathbf{EP}$ | $\frac{-a-c_{1}+bc_{1}+c_{2}-bc_{2}}{2(-1+b)}$ | $\frac{\begin{aligned} -3a+ab-bc_{0}+b^{2}c_{0}-c_{1} \\ +bc_{1}-c_{2}+bc_{2} \end{aligned}}{4(-1+b)}$ | $\frac{-a-c_{0}+bc_{0}}{2(-1+b)}$ |

**Comment (3):** The images in the text are not clear enough. They are blurry, making it difficult to see the numbers and symbols.

**Answer:** Thank you for your constructive comments. We have replaced unclear Fig 5, 6, and 7 with more detailed and accurate representations. The revised figures are presented as follows:

$$\left( a \right)\mathrm{under}no-encroachment$$

$$\left( b \right)\mathrm{under} encroachment$$

**Fig 5. Comparison of equilibrium returns from biomass power plants. （**The change appears on Page 26.**）**

**Fig 6 Impact of different subsidy recipients on social welfare in the no-encroachment scenario. （**The change appears on Page 27.**）**

$$\left( a \right) b=0.4$$

$$\left( b \right) b=0.8$$

**Fig. 7 Impact of different subsidy recipients on social welfare in the encroachment scenario. （**The change appears on Page 29.**）**

**Comment (4):** The format of the references is also messy.

**Answer:** Thank you for your advice and we apologize for our error. We have systematically formatted all cited references throughout the manuscript in accordance with PLOS ONE’s citation guidelines to ensure full compliance with the journal’s requirements.

**Comment (5):** The English expression in the article needs improvement.

**Answer:** Thank you very much for your valuable feedback on the English expression in our article. We fully acknowledge that there is room for improvement in this aspect, and we sincerely appreciate your pointing it out. Upon receiving your comment, we immediately took the following steps to address this issue: We first assigned a team of native English speakers who are also experts in our research field to carefully review the entire manuscript. They have made comprehensive revisions to the language, including grammar, vocabulary, sentence structure, and overall writing style. The evidence for the completion of the English polishing is presented as follows.

In addition, we have also used professional language editing tools to double-check for any remaining errors or awkward expressions. We have made a great deal of effort to ensure that the revised version of the article is more fluent, accurate, and in line with the academic English writing norms.

Once again, thank you for your constructive feedback. We believe that these improvements will significantly enhance the quality of our article and make it more accessible to a wider international audience.

**Response to Reviewer: 2’s Comments**

**Comment:** This manuscript investigates government subsidy strategies within the biomass power supply chain under different scenarios of channel competition and encroachment. Using a game-theoretic model, the authors analyze eight subsidy scenarios and identify conditions that optimize profits and social welfare. Key findings include that subsidies to farmers consistently maximize social welfare, while subsidies to middlemen may harm welfare in high-competition scenarios. The equilibrium suggests that farmers prefer encroachment to induce subsidies.
 The discussion in this paper is complete. If it can be improved in quality, it can be considered for publication.

**Answer:** Thanks for your valuable comments and suggestions. We will answer your questions one by one.

**Comment (1):** It is better to introduce the definition of “standard Stackelberg game setup”. Since there are games, are there strategies? Are strategies fixed or evolutionary?

**Answer:** Thank you for pointing this out. The reviewer is correct, and we have incorporated the definition of the Stackelberg game into the third section of the article and referenced pertinent literature. These changes appear on Page 12. For your convenience, we repeat these modifications here:

***“****This setup follows a standard Stackelberg game framework, where one party assumes the role of a leader and makes an initial decision, while the follower observes this decision before determining its own response [28]. The Stackelberg game is a sequential decision-making model solved through backward induction, widely applied in hierarchical decision-making scenarios [29].****”***

The government’s strategy involves deciding whether to provide subsidies and to whom. Farmers, on the other hand, must decide whether to bypass the intermediary channel by directly supplying straw to biomass power plants. Given that these strategies are realistic, there exists a strategic interaction between the two parties. Specifically, depending on the farmers’ choice to bypass or not bypass the intermediary channel, the government needs to determine the most appropriate recipients of subsidies.

This paper establishes and compares eight decision scenarios based on whether farmers choose to bypass the intermediary channel (N/E) and how the government implements its subsidy strategy (N/M/F/P). Optimal solutions for each scenario are derived. Through this comparison, we identify stable equilibrium strategies for both farmers and the government in certain specific cases. This type of strategic interaction analysis is common in existing research. For instance, Liu [2] analyzed four scenarios under the interaction between an e-commerce platform’s own brand production outsourcing strategy and the OEM channel model: adopting a common contract manufacturer under the agency model (AC), adopting different contract manufacturers under the agency model (AD), adopting a common contract manufacturer under the resale model (RC), and adopting different contract manufacturers under the resale model (RD). The equilibrium prices in these four scenarios are first determined, followed by an explanation of the influence of key parameters on these prices, and finally, a discussion on the impact of production monopoly effects and competition on equilibrium prices. A similar interaction design is used to obtain the equilibrium strategy for a specific scenario.

**Comment (2):** I encourage the authors to discuss why is it necessary to divide eight scenarios. How does this way of dividing between scenarios contribute to the core conclusions of this paper?

**Answer:** Thank you so much for your valuable comments and suggestions. We deeply appreciate your insights, which have provided us with a great opportunity to further clarify and strengthen our research.

We understand your concern regarding the necessity of dividing the eight scenarios in our paper. In fact, this division is of great significance and closely related to the core research of our paper. Based on the existing literature on game theory, in Section Three, we specifically focus on the interaction between government subsidies and farmers’ channel invasion. Different combinations of farmers’ decisions on whether to invade the middleman channel (N/E) and the government departments’ implementation of subsidy strategies (N/M/F/P) [2, 30] lead to a variety of complex interaction situations. By dividing it into eight distinct scenarios, we can systematically and comprehensively analyze these different interaction situations. This allows us to capture the nuances and specific characteristics of each situation, enabling a more in-depth exploration of the relationships and mechanisms at play.

In the fourth section, we present the pricing equilibrium strategies for each of the eight scenarios. This detailed analysis of the equilibrium strategies in different scenarios provides a solid foundation for our subsequent conclusion drawing. In the fifth section, through comparing the equilibrium outcomes of these eight scenarios, we are able to clearly identify the impact of different combinations of farmers’ and government’s decisions on the overall system. For example, we can observe how different subsidy strategies affect farmers’ incentives for channel invasion, and what kind of equilibrium states are achieved under different circumstances. These comparisons and analyses directly contribute to the main conclusions of our paper, enabling us to draw more accurate and comprehensive conclusions about the interaction between government subsidies and farmers’ channel invasion behavior.

In summary, the division of the eight scenarios is not only a necessary step to comprehensively analyze the complex interaction in our research context but also a crucial basis for us to derive the core conclusions of this paper. We believe that this explanation can help you better understand the rationality and significance of our research design.

**Comment (3):** **The format of citations is not consistent**.

**Answer:** We have systematically formatted all cited references throughout the manuscript in accordance with PLOS ONE’s citation guidelines to ensure full compliance with the journal’s requirements.

**Comment (4):** “Scenario NN (Fig a)” and similar later on: which figure?

**Answer:** We were really sorry for our careless mistakes. Thank you for your reminder. We have re-corrected the references for this part of the picture on Page 13. For your convenience, we repeat these modifications here:

*(1). “Scenario NN Fig 1(a)”: There is No channel competition, and the relevant sector does Not subsidize the supply chain members.*

*(2). “Scenario NF Fig 1(b)”: There is No channel competition, and the relevant sector subsidizes the farmers within the supply chain.*

*(3). “Scenario NM Fig 1(c)”: There is No channel competition, and the relevant sector subsidizes the middlemen in the supply chain.*

*(4). “Scenario NP Fig 1(d)”: There is No channel competition, and the relevant sector subsidizes the power plants in the supply chain.*

*(5). “Scenario EN Fig 1(e)”: Farmers opt to establish new supply channels, and the relevant sector does Not subsidize the supply chain members.*

*(6). “Scenario EF Fig 1(f)”: Farmers opt to establish new supply channels, and the relevant sector subsidizes the farmers in the supply chain.*

*(7). “Scenario EM Fig 1(g)”: Farmers opt to establish new supply channels, and the relevant sector subsidizes the middlemen in the supply chain.*

*(8). “Scenario EP Fig 1(h)”: Farmers opt to establish new supply channels, and the relevant sector subsidizes the power plants in the supply chain.*

**Fig 1. Eight scenarios in which the government subsidizes members of the biomass power supply chain.**

**Comment (5):** An optional suggestion: change “There is no channel competition, and the relevant sector does not subsidize the supply chain members” to “There is No channel competition, and the relevant sector does Not subsidize the supply chain members” and similar later on, would make it easier for readers.

**Answer:** We sincerely thank the reviewer for careful reading. As suggested by the reviewer, we have corrected the “There is no channel competition, and the relevant sector does not subsidize the supply chain members” into “*There is No channel competition, and the relevant sector does Not subsidize the supply chain members*”. Furthermore, identical revisions were applied consistently throughout the paragraph.

**Comment (6):** “similar to Jiang et al.’s study”. You missed a citation.

**Answer:** We sincerely regret our oversight. We appreciate your timely reminder. We have addressed the missing citations and established the unit costs for each channel by referencing existing literature. These changes appear on Page 14 :”*In addition, the unit processing cost of biomass in different sales channels is denoted as* $c_{0}/c_{1}{/c}_{2}{/c}_{3}$ *for farmers, middlemen, and power plants, respectively [31].*”

**Comment (7):** Please write more in figure captions, making a figure self-contained.

**Answer:** Following your recommendations, we revised the captions of several images in the text to enhance their comprehensiveness.

$$\left( a \right) under no-encroachment$$

$$\left( b \right) under encroachment$$

**Fig 3. Comparative chart of farmers' equilibrium returns. (**These changes appear on Page 24.**)**

$$\left( a \right) under no-encroachment$$

$$\left( b \right) under encroachment$$

**Fig 4. Comparative chart of middlemen's equilibrium returns. (**These changes appear on Page 25.)

$$\left( a \right) under no-encroachment$$

$$\left( b \right) under encroachment$$

**Fig 5. Comparison of equilibrium returns from biomass power plants. (**These changes appear on Page 26.)

**Comment (8):** Punctuate each independent formula to ensure sentences have an end.

**Answer:** Thank you for your meticulous review and for highlighting the issue concerning the punctuation of independent formulas in our manuscript. We fully recognize the significance of correct punctuation in ensuring that our sentences are both complete and easily comprehensible, particularly when dealing with independent formulas. Consequently, appropriate punctuation marks have been added to all independent formulas throughout the manuscript, thereby enhancing the overall completeness and readability of each sentence. Such on Pages 14-15: “*In addition, the unit processing cost of biomass in different sales channels is denoted as* $c_{0}/c_{1}{/c}_{2}{/c}_{3}$ *for farmers, middlemen, and power plants, respectively [31]. The demand function [1]：*

$$\begin{aligned} d_{1}^{N}=a-p_{1},\#\text{(1)} \end{aligned}$$

$$\begin{aligned} d_{1}^{E}=a-p_{1}+bp_{2},\#\text{(2)} \end{aligned}$$

$$\begin{aligned} d_{2}=a-p_{2}+bp_{1}.\#\text{(}\text{3}\text{)}\text{”} \end{aligned}$$

*The profit of members in the biomass power generation supply chain is denoted as* $\pi_{i}(a=F;M;P$*), where F represents farmers, M represents middlemen, and P represents power plants. In existing literature, consumer surplus is commonly defined as “the additional benefit or surplus that consumers derive from market transactions above the price paid [32, 33].” The consumer surplus function [34]：*

$$\begin{aligned} CS=\frac{\left( d_{1}+d_{2} \right)^{2}}{2}.\#\text{(4)} \end{aligned}$$

*The social welfare（the combined benefits of all stakeholders in the biomass power generation supply chain, including both producer profits and consumer surplus [35]） formula is:*

$$\begin{aligned} SW=CS+\pi_{F}+\pi_{M}+\pi_{P}.\#\text{(5)}\text{”} \end{aligned}$$

**Comment (9):** There are many non-English punctuation marks.

**Answer:** Thank you for pointing out the issue of non-English punctuation marks in our manuscript. We are truly sorry for this oversight and understand that it can affect the overall quality and professionalism of our work. We will use text editing tools that can help detect such punctuation errors more efficiently. After the corrections are made, we will conduct a final check to make sure that no non-English punctuation marks remain in the manuscript.

**Comment (10):** I did not see the definitions of CS and SW.

**Answer:** Thank you for highlighting the lack of definitions for “CS” and “SW” in our manuscript. We sincerely apologize for this oversight, as we understand the importance of clearly defining all abbreviations and terms to ensure the clarity and comprehensibility of our article.

Upon reading your comment, we immediately realized the need to rectify this issue. We have now added the definitions of “CS” and “SW” at their first occurrences in the text. Specifically, “CS” denotes Consumer Surplus, defined as the incremental benefit or surplus that consumers derive from market transactions[32, 33]. “SW” represents Social Welfare, which encompasses the combined benefits of all stakeholders, including both producer profits and consumer surplus[35]. We have also double-checked the entire manuscript to ensure that all other abbreviations and technical terms are properly defined. This will help readers, especially those who may not be fully familiar with our specific research area, to better understand the content of our article.

**Comment (11):** Phrases like “the Wu [23] article” are weird.

**Answer:** We appreciate your suggestion. The original statement indeed appeared weird within the text. Consequently, we have removed similar expressions, directly incorporated references immediately following the relevant viewpoints, and revised problematic content throughout the entire document.

**Comment (12):** this “chapter”. There is no chapter in a journal article, only sections.

**Answer:** Thank you for your astute observation regarding the incorrect usage of the term “this chapter” in our journal article. We deeply appreciate your attention to such details, as it is essential for maintaining the accuracy and professionalism of our work.

We acknowledge that in the context of a journal article, the appropriate term to use is “this section” rather than “chapter”. We made an oversight into our writing, and we have already taken steps to correct all instances of this error throughout the manuscript.

Our team has carefully reviewed the entire article to ensure that all references to the different parts of the article are correctly labeled as “sections”. This will help to avoid any confusion for readers and ensure that our writing adheres to the standard conventions of journal article formatting.

**Comment (13):** Table 2: I did not see the definitions of W1 and P1. What is the difference between W and w? What is the difference between P and p?

**Answer:** Thank you for your suggestion. In the text, $w_{1}$ denotes the unit wholesale price of biomass raw materials sold by farmers to middlemen, while $p_{1}$denotes the unit retail price transferred from middlemen to biomass power plants. Due to an input error in the formula, both uppercase and lowercase letters such as *“*$P$*”, “*$p$*”, “*$W$*”,* and *“*$w$*”* appeared simultaneously in the text. Under normal circumstances, these variables should all be in lowercase to maintain consistency. For clarity, the parameter symbols and descriptions are listed in Table 2.

Table 2 Symbols. (These changes appear on Page 12.)

| notation | hidden meaning |
| --- | --- |
| $w_{1}$ | Wholesale prices of farmers selling to middlemen |
| $p_{1}$ | The unit price at which the middleman sells to the power plant |
| $p_{2}$ | The unit price at which the farmer sells to the power plant |
| $p_{0}$ | Unit tariffs for biomass power plants |
| $t$ | Conversion factor between straw supply and electricity generation |
| $d_{1}$ | Demand for channel 1 |
| $d_{2}$ | Demand for channel 2 |
| $d_{1}^{N}$ | Demand for channel 1 in case of non-encroachment |
| $d_{1}^{E}$ | Demand for channel 1 at the time of the encroachment |
| $c_{0},c_{1},c_{2}{,c}_{3}$ | transportation costs at all stages.$c>0$ |
| $b$ | the intensity of competition between channels.$1>b>0$ |
| $a$ | Demand for biomass feedstock. |
| $CS$ | Consumer surplus |
| $SW$ | Social welfare |
| $m$ | Government subsidized unit *prices* for biomass feedstocks $m>0$ |

**Comment (14):** Font sizes in figures are unacceptable.

**Answer:** Thank you for bringing this issue regarding the font sizes in the figures to our attention. We understand that the current font sizes in the figures are not in an acceptable state and can undermine the clarity and readability of our presented data. Immediately upon reading your comment, we have initiated the process of rectifying this problem. Specifically, we adjusted the font size of the numbers in the tables and formulas and replaced the low-resolution images. To ensure that the font size of the numbers within the text adheres to the journal’s specified standards. Such as: “*Using the inverse solution method: first let (16) be the first order derivative of the*$p_{1}$ *take the first order derivative to obtain the reaction function* $p_{1}=\frac{1}{2}(a+c_{2}+w_{1})$ *to obtain the reaction function. Bringing this reaction function into (17) and then letting (17) take the first order derivative with respect to the*$w_{1}$ *find the first-order derivative, we can get*$w_{1}=\frac{1}{2}(a+c_{1}-c_{2})$ *. Bringing*$w_{1}=\frac{1}{2}(a+c_{1}-c_{2})$ *into the above*$p_{1}=\frac{1}{2}(a+c_{2}+w_{1})$ *to get the equilibrium price. For convenience, we put the equilibrium pricing results in Table 3*.”

**Table 3 Equilibrium pricing in the no-encroachment channel scenarios.**

| **Scenario** | $\boldsymbol{w}_{\mathbf{1}}$ | $\boldsymbol{p}_{\mathbf{1}}$ |
| --- | --- | --- |
| $\mathbf{NN}$ | $\frac{1}{2}(a+c_{1}-c_{2})$ | $\frac{1}{4}(3a+c_{1}+c_{2})$ |
| $\mathbf{NF}$ | $\frac{1}{2}(a+c_{1}-c_{2}-m)$ | $\frac{1}{4}(3a+c_{1}+c_{2}-m)$ |
| $\mathbf{NM}$ | $\frac{1}{2}(a+c_{1}-c_{2}+m)$ | $\frac{1}{4}(3a+c_{1}+c_{2}-m)$ |
| $\mathbf{NP}$ | $\frac{1}{2}(a+c_{1}-c_{2})$ | $\frac{1}{4}(3a+c_{1}+c_{2})$ |

**Comment (15):** English proofreading and polishing are needed. All kinds of formats need to be error-free. Any error will influence the acceptance of the manuscript.

**Answer:** Thank you so much for pointing out the need for English proofreading, polishing, and format correction. We fully recognize the importance of these aspects in ensuring the quality of our manuscript and understand that any errors can have a negative impact on its acceptance.

Upon receiving your comment, we immediately acted. We have assigned a team of native English speakers with expertise in academic writing to carefully proofread the entire manuscript. They will check for grammar, spelling, punctuation, and vocabulary errors, as well as improve the overall flow and clarity of the language. The evidence for the completion of the English polishing is presented as follows.

In addition, we also use professional language editing tools to double-check for any remaining issues.

Regarding the format, we have reviewed all the sections of the manuscript, including the title page, abstract, main text, references, and any supplementary materials, to ensure that they comply with the specified formatting requirements. We have meticulously checked for consistent use of fonts, line spacing, margins, and citation styles.

**Response to Reviewer: 3’s Comments**

**Comment:** The abstract is dense, making it difficult to discern the key conclusions and appreciate the utility of the findings. The discussion of subsidies could be more concise. Secondary elements, such as competition intensity and equilibrium results, dilute the main theme.

**Answer:** Thank you very much for your detailed and valuable feedback. We sincerely appreciate your careful review of our work.

Based on your valuable comments, we have modified the presentation of some of the conclusions in the abstract: “*Notably, the analysis indicates that under the encroachment scenario, government subsidies directed solely to middlemen may negatively affect the overall social welfare of the biomass power generation supply chain. Furthermore, as channel competition intensifies, the probability and extent of this negative impact on social welfare are likely to increase. Additionally, the equilibrium outcome of the game-theoretic model establishes that farmers will invariably choose encroachment as a means to trigger government subsidies, thereby maximizing their profits. These findings provide essential theoretical insights into farmers' strategic behavior aimed at income enhancement and offer guidance for government subsidy policies to achieve optimal social welfare.*”

**Comment (1):** Condense some contextual details to focus on the specific gaps in research related to channel encroachment and government subsidies and include a clear thesis statement, summarizing the research’s contribution.

**Answer:** Thank you for your valuable feedback. We fully understand and appreciate your suggestion regarding the need to condense contextual details and enhance the clarity of our research focus and thesis statement.

In response to your comment, we have taken the following steps in the revised manuscript. Firstly, we restructure the literature review into a distinct second section, separating it from the introduction. Secondly, this study incorporates additional research background concerning biomass channel invasion and government subsidies. These changes appear on Pages 5-6. For your convenience, we repeat these modifications here: “*Conversely, in the biomass raw material supply sector, farmers are increasingly choosing to bypass middlemen [10] and supply raw materials directly to power plants [11]. For example, in Gongzhuling City, Jilin Province, following the autumn harvest, farmers transport packaged straw directly to Guoneng Gongzhuling Biomass Power Generation Co., Ltd. [36]. Similarly, in Tengzhou City, Shandong Province, harvested wheat straw is baled and sold directly to the local biomass power plant by farmers [37]. Existing case studies indicate that the expansion of biomass raw material supply channels by farmers may also be influenced by government subsidies. For instance, the "Notice on the Subsidy Funds for the Comprehensive Utilization of Rice Straw in 2022" issued in Qingpu District, Shanghai, specifies that entities purchasing rice straw and implementing straw utilization away from fields within the district are eligible for subsidies of 300 RMB/ton, with the city contributing 240 RMB/ton and the district providing 60 RMB/ton. Additionally, all business units and enterprises involved in the comprehensive utilization management of straw across the province receive a subsidy of 20 RMB per ton based on the quantity of corn and rice straw utilized [38]. These examples highlight the significant role of government subsidies in facilitating the expansion of farmers' supply channels. However, the interaction between government subsidies and channel expansion remains an area that has received limited scholarly attention.”* Thirdly, we have added a dedicated section in the introduction and literature review parts of the manuscript. In the introduction, we systematically restructured and refined the research contributions of this article: “*This study makes several contributions to existing literature. First, to the best of current knowledge, it is the first to investigate government subsidy strategies in biomass power supply chains consisting of farmers and middlemen under horizontal competition. Previous research has primarily focused on competition among vertically related members. Second, this study derives pricing equilibrium strategies for different stakeholders under various government subsidy schemes, yielding several notable findings. Unlike scenarios without encroachment, where subsidies consistently enhance social welfare, subsidizing middlemen in the presence of encroachment may negatively impact social welfare. Furthermore, the findings suggest that under intensified channel competition, a lower subsidy to middlemen increases the probability of a decline in social welfare.*” By doing so, we have demonstrated the novelty and significance of our study and have provided a more comprehensive understanding of the research topic.

Furthermore, we have streamlined the introduction by removing redundant information and reorganizing its structure to enhance clarity. The revised organization of the first part is as follows: The initial paragraph provides an overview of the research background, emphasizing that biomass power generation is a critical component of sustainable energy development and highlighting its potential for further advancement. The subsequent paragraph elucidates that governments worldwide commonly employ policies to regulate the decisions of biomass supply chain participants, thereby fostering the sustainable development of the biomass power generation supply chain, which sets the stage for the research focus of this paper. The third paragraph underscores the pivotal role of government subsidies in guiding channel invasion within the biomass supply chain, while noting that existing literature lacks comprehensive analysis of the interplay between these two elements. The fourth paragraph delineates the primary issues addressed in this study. Finally, the fifth paragraph encapsulates the research contributions of this paper.

**Comment (2):** Some general findings are repeated without directly connection to the study’s objectives. Add a explicit comparison of how this study differs from others.

**Answer:** Thank you for your astute observation and valuable feedback. We deeply appreciate your input, which has highlighted areas where our manuscript can be significantly improved.

We acknowledge that there are instances where some general findings have been presented without a clear and direct connection to our study's objectives. This oversight was unintentional, and we have taken immediate steps to address it. In the revised version of the manuscript, we have carefully reviewed each section and ensured that all findings are directly relevant to our research questions and objectives. Any redundant or unrelated information has been removed, and the remaining content has been reorganized to enhance clarity and coherence.

Regarding your suggestion to add an explicit comparison of how our study differs from others, we fully agree that this is an important aspect that was lacking in the original submission. To address this, in the literature review, we have provided a more comprehensive review of the existing literature on the topic, highlighting the key studies and their findings. The comparison of our paper with the extant literature is shown in Table 1.

**Table 1 . Comparison with the literature.**

|  | | **Incentive policy** | | | | | | | **Channel** | | | | | **Policy target** | | | | | | | | | | |
| --- | --- | --- | --- | --- | --- | --- | --- | --- | --- | --- | --- | --- | --- | --- | --- | --- | --- | --- | --- | --- | --- | --- | --- | --- |
|  | | **Taxes** | | | **Quota**  **system** | | **Subsidization** | | | **Encroachment** | | | **Non-encroachment** | | | | **Farmer** | | | **Middleman** | | | **Manufacturer** | |
| **[12]** |  | |  | | | **✓** | | | |  | **✓** | | | | **✓** | | |  | | |  | | |  |
| **[13]** |  | |  | | | **✓** | | | | **✓** |  | | | |  | | |  | | | **✓** | | |  |
| **[14]** |  | | **✓** | | | **✓** | | | |  | **✓** | | | | **✓** | | |  | | | **✓** | | |  |
| **[15]** | **✓** | |  | | |  | | | |  | **✓** | | | |  | | |  | | | **✓** | | |  |
| **[16]** |  | |  | | | **✓** | | | |  | **✓** | | | |  | | |  | | | **✓** | | |  |
| **[17].** |  | | **✓** | | | **✓** | | | | **✓** |  | | | |  | | | **✓** | | | **✓** | | |  |
| **[18]** |  | | **✓** | | | **✓** | | | |  | **✓** | | | | **✓** | | |  | | | **✓** | | |  |
| **[19]** |  | |  | | | **✓** | | | |  | **✓** | | | |  | | | **✓** | | | **✓** | | |  |
| **[20]** |  | | **✓** | | | **✓** | | | |  | **✓** | | | | **✓** | | | **✓** | | | **✓** | | |  |
| **[21]** |  | |  | | | **✓** | | | |  | **✓** | | | | **✓** | | | **✓** | | |  | | |  |
| **[22]** |  | |  | | | **✓** | | | |  | **✓** | | | | **✓** | | | **✓** | | | **✓** | | |  |
| **[23]** |  | |  | | | **✓** | | | |  | **✓** | | | | **✓** | | | **✓** | | | **✓** | | |  |
| **[24]** |  | |  | | |  | | | | **✓** |  | | | |  | | |  | | |  | | |  |
| **[25]** |  | |  | | | **✓** | | | | **✓** |  | | | | **✓** | | |  | | |  | | |  |
| **[26]** |  | |  | | | **✓** | | | |  | **✓** | | | | **✓** | | | **✓** | | | **✓** | | |  |
| **[27]** |  | |  | | |  | | | | **✓** |  | | | |  | | |  | | |  | | |  |
| **This paper** | |  | |  | | | | **✓** | | **✓** | | **✓** | | | | **✓** | | | **✓** | | | **✓** | |  |

**Comment (3):** The assumption of linear subsidies and static competition intensity limit the model’s applicability to dynamic markets. Incorporating uncertainties like fluctuating biomass prices would enhance the robustness of the model.

While the model can predict outcomes, it could provide some actionable insights for policymakers.

**Answer:** Thank you very much for your insightful and constructive comments. We wholeheartedly appreciate your feedback regarding the limitations of our model and the valuable suggestions for improvement. In practice, governments across the globe commonly employ linear subsidies as a policy tool to address the challenges of sustainable supply chain development [39, 40]. In constructing the model presented in this article，we adopted the assumption that the subsidy follows a linear relationship [41-43].

In this paper, the parameter $b$ ($0\leq b\leq1$) quantifies the competitive intensity between the two channels. In Section Five, we compare the equilibrium profits of farmers, middlemen, and biomass power plants under conditions of high competitive intensity $b \geq0.5$ versus low competitive intensity（$b ＜ 0.5$）, and analyze the effects of varying channel competition on social welfare. The study focuses on biomass prices, specifically the wholesale price w1 and the unit selling prices p1 and p2 for each channel. We formulate profit functions for farmers, middlemen, and biomass power plants across eight scenarios and derive the corresponding equilibrium prices for each scenario. These prices are fixed within their respective scenarios.

As you suggested, incorporating uncertain factors can enhance the robustness of the model. The focus of this research is to examine the strategic interaction between government subsidies and channel competition intensity, particularly in scenarios with and without channel invasion. The dynamic extension of subsidies is beyond the scope of this study. We anticipate conducting further investigations in subsequent studies. Consequently, we have made specific adjustments to our future research directions.

Following your recommendations, we have reorganized and enriched our management implications to provide more actionable suggestions for policymakers. These changes appear on Pages 32-33. For your convenience, we repeat these modifications here:

“*（1）Whether supply channel competition exists or not, government subsidies for farmers are crucial for ensuring the stable supply of biomass raw materials. In practice, farmers often exhibit low willingness to supply straw and frequently resort to field burning, leading to resource wastage [44]. The government can leverage subsidy policies to address this issue. Firstly, it can encourage biomass power generation enterprises to establish long-term and stable cooperative relationships with farmers by providing additional subsidies for joint projects. This promotes information sharing and collaborative operations between upstream and downstream supply chain partners, thereby enhancing overall supply chain efficiency. Secondly, policy preferences and financial support can be offered to farmers who actively participate in building the coordination mechanism of the biomass power generation supply chain. This fosters a favorable market environment and enhances supply chain stability. （2）Interestingly, our research demonstrates that the government should exercise caution when providing subsidies to intermediaries. In the absence of external competition, subsidies can effectively lower operational costs for intermediaries, thereby ensuring they remain motivated to organize and coordinate the supply of biomass raw materials, thus enhancing supply stability. However, in the presence of external competition, the government must carefully consider the level of subsidies provided to intermediaries. Poorly designed subsidies may exacerbate channel competition. （3）When the intensity of channel competition is high, an excessively low subsidy coefficient fails to adequately incentivize key links in the biomass power generation supply chain. This leads to unstable raw material supply, which negatively impacts the production efficiency of biomass power generation enterprises and ultimately reduces social welfare. Additionally, an overly low subsidy coefficient may result in cost transfers across the supply chain, causing increases in the price of biomass power generation products and reductions in consumer surplus, further diminishing social welfare. Particularly, when subsidies are provided to intermediaries under such conditions, it may exacerbate unnecessary price competition. It is recommended that the government, considering long-term perspectives and incorporating the findings of this study, develop forward-looking subsidy policies.”*

**Comment (4):** The conclusions could link findings to existing studies to highlight the contributions. Including a figure could enhance the presentation of key findings. The conclusions could be condensed to improve readability.

**Answer:** Thank you so much for your insightful and constructive comments regarding the conclusions section of our manuscript. We truly appreciate your suggestions as they will help us to significantly enhance the quality and impact of our work.

In the revised version, we carefully revisited the conclusions and added realistic data that compare and contrast our results with those of previous studies. The existing conclusion has been refined, resulting in a revised version on Page 31: “*This study examined eight strategic scenarios within the biomass power generation supply chain, leading to the following key conclusions. First, equilibrium prices for supply chain participants are significantly affected by government subsidy strategies and channel encroachment behaviors. Specifically, regardless of whether encroachment occurs, subsidies directed toward farmers or intermediaries reduce the raw material procurement cost for biomass power plants, with subsidies to farmers yielding the lowest supply price for biomass raw materials [42]. Second, the analysis demonstrates that in the absence of encroachment, subsidizing upstream supply chain members, such as intermediaries and farmers, maximizes social profits. However, contrary to existing theories [45], the findings indicate that under encroachment conditions, inadequate government subsidy intensity may negatively impact social profits. Therefore, government agencies must strategically determine subsidy recipients and levels based on the degree of channel competition. This study provides a new perspective on government incentive strategies for biomass power supply chains and offers practical guidance for policymakers responsible for subsidy allocation. Finally, the research findings suggest that farmers benefit from establishing direct sales channels, while government subsidies play a crucial role in supporting these transitions. This strategy mitigates the loss of intermediary profits by increasing farmers' earnings through higher direct sales prices. For example, in Bozhou, Anhui Province, the straw purchase price from middlemen is about 240 yuan per ton, whereas direct sales to power plants yield around 290 yuan per ton [46]. Additionally, government subsidies enhance farmers' willingness to supply biomass [47]. In Ketian Town, Yanhe County, Guizhou Province, a subsidy of 200 yuan per ton for straw has resulted in an expected recovery of 1,900 tons of straw in 2024, generating an estimated total output value of 600,000 yuan [48].*”

We believe that these changes will make our manuscript more focused and will effectively communicate the significance and novelty of our research. Once again, thank you for your constructive feedback. We look forward to your further evaluation of the revised version.

Sincerely,

Guangyin Xu

**Reference**

1.	Wang T-Y, Li Y-L, Yang H-T, Chin K-S, Wang Z-Q. Information sharing strategies in a hybrid-format online retailing supply chain. International Journal of Production Research. 2021;59(10):3133-51.

2.	Liu B, Liu P, Shen YJAoOR. Contract manufacturer encroachment and its impact on OEM’s sales mode and social welfare in a platform-based supply chain. 2024:1-24.

3.	Vazifeh Z, Mafakheri F, An CJSc, society. Biomass supply chain coordination for remote communities: A game-theoretic modeling and analysis approach. 2021;69:102819.

4.	Wei J, Wang Y, Lu J. Information sharing and sales patterns choice in a supply chain with product’s greening improvement. Journal of Cleaner Production. 2021;278:123704.

5.	Yoro KO, Daramola MO. CO2 emission sources, greenhouse gases, and the global warming effect. Advances in carbon capture: Elsevier; 2020. p. 3-28.

6.	Wang R, Li H, Cai W, Cui X, Zhang S, Li J, et al. Alternative pathway to phase down coal power and achieve negative emission in China. 2022;56(22):16082-93.

7.	Abdul Malek A, Hasanuzzaman M, Rahim NAJCT, Policy E. Prospects, progress, challenges and policies for clean power generation from biomass resources. 2020;22:1229-53.

8.	Wang ZW, Xu GY, Wang ZF, Zhang ZP. Sustainability of agricultural waste power generation industry in China: criteria relationship identification and policy design mechanism. Environment Development and Sustainability. 2022;24(3):3371-95. doi: 10.1007/s10668-021-01570-2. PubMed PMID: WOS:000659835800002.

9.	Amiri-Pebdani S, Alinaghian M, Khosroshahi HJEP. Pricing in competitive energy supply chains considering government interventions to support CCS under cap-and-trade regulations: A game-theoretic approach. 2023;179:113630.

10.	Giannoccaro G, de Gennaro BC, De Meo E, Prosperi MJEE. Assessing farmers' willingness to supply biomass as energy feedstock: cereal straw in Apulia (Italy). 2017;61:179-85.

11.	Zyadin A, Natarajan K, Chauhan S, Singh H, Hassan MK, Pappinen A, et al. Indian farmers’ perceptions and willingness to supply surplus biomass to an envisioned biomass-based power plant. 2015;6(1):42-54.

12.	Wang Z, Wang Z, Tahir N, Wang H, Li J, Xu G. Study of synergetic development in straw power supply chain: Straw price and government subsidy as incentive. Energy Policy. 2020;146:111788. doi: <https://doi.org/10.1016/j.enpol.2020.111788>.

13.	Ma N, Huang Z, Qi Y. Simulation Study on Complex Systems of Forest Biomass Power Generation Supply Chain in China. Computational Intelligence and Neuroscience. 2022;2022. doi: 10.1155/2022/7202352. PubMed PMID: WOS:000793535600002.

14.	Ghani NMAMA, Vogiatzis C, Szmerekovsky JJEP. Biomass feedstock supply chain network design with biomass conversion incentives. 2018;116:39-49.

15.	Liu LW, Ye JH, Zhao YF, Zhao ED. The plight of the biomass power generation industry in China - A supply chain risk perspective. Renewable & Sustainable Energy Reviews. 2015;49:680-92. doi: 10.1016/j.rser.2015.04.151. PubMed PMID: WOS:000357141900055.

16.	Li YA, Lin J, Qian YJ, Li DH. Feed-in tariff policy for biomass power generation: Incorporating the feedstock acquisition process. European Journal of Operational Research. 2023;304(3):1113-32. doi: 10.1016/j.ejor.2022.05.011. PubMed PMID: WOS:000862712400004.

17.	Rajabzadeh H, Babazadeh R. A game-theoretic approach for power pricing in a resilient supply chain considering a dual channel biorefining structure and the hybrid power plant. Renewable Energy. 2022;198:1082-94. doi: <https://doi.org/10.1016/j.renene.2022.08.118>.

18.	He N, Jiang Z-Z, Huang S, Li KJIJoPR. Evolutionary game analysis for government regulations in a straw-based bioenergy supply chain. 2023;61(18):6093-114.

19.	Lin J, Li Y, Qian Y, Liu G. Effectiveness of government subsidies for biomass-based industries: Incorporating the feedstock acquisition process. Energy Policy. 2024;192:114251. doi: <https://doi.org/10.1016/j.enpol.2024.114251>.

20.	Jiang ZZ, He N, Xiao L, Sheng Y. Government subsidy provision in biomass energy supply chains. Enterprise Information Systems. 2019;13(10):1367-91. doi: 10.1080/17517575.2019.1658807. PubMed PMID: WOS:000486068800002.

21.	Wang L, Watanabe TJE. A stackelberg game theoretic analysis of incentive effects under perceived risk for China’s straw-based power plant supply chain. 2016;9(6):455.

22.	Wu J, Zhang J, Yi W, Cai H, Li Y, Su ZJE. A game-theoretic analysis of incentive effects for agribiomass power generation supply chain in China. 2021;14(3):546.

23.	Wang Z, Hou T, Wang ZJW, Valorization B. Revenue Coordination of Straw Power Generation Supply Chain Under Carbon Trading Environment. 2023;14(5):1769-85.

24.	Qinliang T, Yanming D, Xingping ZJJoLU. A study of farmers’ willingness and behavior in the comprehensive utilization of agricultural straw. 2014;42(5):106-10.

25.	Luo K, Zhang X, Tan Q. A Co-Opetition Straw Supply Strategy Integrating Rural Official Organizations and Farmers’ Behavior in China. Energies [Internet]. 2018; 11(10).

26.	Fan K, Li XN, Wang L, Wang M. Two-stage supply chain contract coordination of solid biomass fuel involving multiple suppliers. Computers & Industrial Engineering. 2019;135:1167-74. doi: 10.1016/j.cie.2019.01.016. PubMed PMID: WOS:000482244100087.

27.	Sun J, Lin J, Qian Y. Game-theoretic analysis of competitive agri-biomass supply chain. Journal of Cleaner Production. 2013;43:174-81. doi: <https://doi.org/10.1016/j.jclepro.2012.12.026>.

28.	Xie J, Wei JCJEJoOR. Coordinating advertising and pricing in a manufacturer–retailer channel. 2009;197(2):785-91.

29.	Wahab OA, Bentahar J, Otrok H, Mourad AJESwA. A Stackelberg game for distributed formation of business-driven services communities. 2016;45:359-72.

30.	Liu P, Yang X, Zhang R, Liu BJC, Engineering I. OEM’s sales formats under e-commerce platform’s private-label brand outsourcing strategies. 2022;173:108708.

31.	Jiang Z-Z, He N, Huang S. Contracting with asymmetric information under government subsidy programmes in a bioenergy supply chain. International Journal of Production Research. 2021;60(18):5571-94. doi: 10.1080/00207543.2021.1966541.

32.	Zhou W, Zhang H, Gan Y, Dai R, Li CJAoOR. Introducing fringe retailer to confront dominant retailer with personalized pricing. 2025:1-26.

33.	Adhikari A, Basu S, Avittathur BJJoCP. Greening Innovation, Advertising, and Pricing Decisions under Competition and Market Coverage. 2025:144951.

34.	Zhou Y, Hu F, Zhou ZJJocp. Pricing decisions and social welfare in a supply chain with multiple competing retailers and carbon tax policy. 2018;190:752-77.

35.	Osorio A, Zhang MJJoCP. Incentivizing environmental investments: The contest-based subsidy allocation mechanism. 2022;380:135132.

36.	Agency XN. "Firewood sticks" turn into "hot commodities". Jilin finds a way out for straw. 2016 December 7th. Available from: Available from: <https://www.gov.cn/xinwen/2016-12/07/content_5144839.htm#1>.

37.	Network CN. Tengzhou, Shandong province: Increase wheat straw recycling efforts to turn waste into treasure 2023 June 9th. Available from: Available from: <https://www.chinanews.com.cn/tp/2023/06-09/10022167.shtml#>.

38.	Agricultural and Rural Affairs Committee of Qingpu District S. Notice on the Allocation of Subsidy Funds for Comprehensive Utilization of Rice Straw in 2022 2024 March 21th. Available from: Available from:<https://www.shqp.gov.cn/agri/nwzwgk/ml/yw/20240402/1165202.html>.

39.	Laborde D, Mamun A, Martin W, Piñeiro V, Vos RJNc. Agricultural subsidies and global greenhouse gas emissions. 2021;12(1):2601.

40.	Chen J-Y, Dimitrov S, Pun HJO. The impact of government subsidy on supply Chains’ sustainability innovation. 2019;86:42-58.

41.	Cai Z, Ye F, Xie Z, Zhang L, Cui TJJoCP. The choice of cooperation mode in the bioenergy supply chain with random biomass feedstock yield. 2021;311:127587.

42.	Wang Z, Wang Z, Tahir N, Wang H, Li J, Xu GJEP. Study of synergetic development in straw power supply chain: Straw price and government subsidy as incentive. 2020;146:111788.

43.	Li Y, Lin J, Qian Y, Li DJEJoOR. Feed-in tariff policy for biomass power generation: Incorporating the feedstock acquisition process. 2023;304(3):1113-32.

44.	Hu J, Lei T, Wang Z, Yan X, Shi X, Li Z, et al. Economic, environmental and social assessment of briquette fuel from agricultural residues in China–A study on flat die briquetting using corn stalk. 2014;64:557-66.

45.	Xue C, Wang XJPe. Study on government subsidy decision-making of straw power generation supply chain. 2017;174:211-8.

46.	News BE. Bozhou: The "Counterattack Road" of a Straw 2023 June 6th. Available from: Available from:<http://ah.anhuinews.com/bozhou/news/yw/202306/t20230606_6900174.html>.

47.	Watanabe R, Watanabe TJJoCP. Factors affecting farmers' risk perceptions regarding biomass supply: A case study of the national bioenergy industry in northeast China. 2016;139:517-26.

48.	County TCOoY. Yanke Town, along the river: Straw recovery turns into "treasure", helping farmers increase income and promoting development 2024 September 2th. Available from: Available from:<https://yhwenming.gog.cn/wmcj045/20240902/20240902_529568.shtml>.
